# Symptom measures in pediatric narcolepsy patients: a review

**DOI:** 10.1186/s13052-021-01068-7

**Published:** 2021-06-02

**Authors:** Hui Ouyang, Xuguang Gao, Jun Zhang

**Affiliations:** Department of Neuromedicine, Peking University People’s Hospital, 11 Xizhimen South Street, Beijing, China

**Keywords:** Questionnaire, Pediatrics, Symptom, Complication, Narcolepsy

## Abstract

**Purpose:**

This study aimed to provide a summary of the measures to assess narcoleptic symptoms or complications in pediatric narcolepsy patients.

**Methods:**

We searched in the National Center for Biotechnology Information (NCBI) for measures of narcoleptic symptoms for pediatric patients. Further review was conducted if relevant questionnaires or information were mentioned.

**Results:**

There were only two narcolepsy-specific questionnaires, the narcolepsy severity scale and Ullanlinna Narcolepsy Scale, neither of them was developed or validated in the pediatric population. For cataplexy, all the measures were study-specific diaries and were not validated questionnaires. For excessive daytime sleepiness, the Epworth Sleepiness Scale was most frequently used to measure excessive daytime sleepiness in children. For nighttime sleep, the Children’s Sleep Habits Questionnaire was most frequently used. For depression, the Children Depression Inventory was the most frequently used. For attention-deficit/hyperactivity disorder, the Child Behavior Checklist was the most frequently used. For quality of life, KIDSCREEN was most frequently used.

**Conclusions:**

At present, there is a lack of disease-specific and validated questionnaires for pediatric narcoleptic patients. This need can be met by modifying and adjusting the existing adult questionnaires and developing new questionnaires for pediatric narcoleptic patients.

## Introduction

Narcolepsy is a life-long neurological disorder with onset primarily during childhood and adolescence, [1] characterized by excessive daytime sleepiness (EDS), cataplexy, sleep paralysis, and hallucinations [2].

The two main symptoms of narcolepsy are EDS, with attacks of irresistible sleep, and cataplexy, defined as “generally brief, sudden episodes of loss of bilateral muscle tone with retained consciousness” often triggered by strong emotions [3]. Noteworthy, apart from the classical tetrad of symptoms, patients with narcolepsy also suffer from other symptoms, including disrupted nocturnal sleep, mood disorder, behavioral and attention disorders [4]. Among other narcolepsy symptoms, the reported prevalence is approximately 40–60% for hypnopompic or hypnagogic hallucinations, 30–50% for sleep paralysis, and about 80% of patients report disrupted nighttime sleep [5, 6]. In adolescents and children, narcolepsy is also strongly related to psychiatric complications, such as mood disorder and attention-deficit/hyperactivity disorder (ADHD), [7, 8] the irritability and motor restlessness of narcolepsy children even mimick the symptom of ADHD [9]. Living with narcolepsy is also related to a lower quality of life, at the same time, it may have a substantial socioeconomic impact [10, 11].

Recently, approaches to the management of pediatric narcolepsy are mostly symptomatically driven, appropriate long-term management requires measures to monitor symptom severity and their changes following treatment, as well as monitor the severity of complications. A variety of subjective tests have been used to measure the severity of symptoms as well as its response to treatment [12–14]. However, these instruments were mostly designed for the adult patients, and may not be appropriate for the pediatric patients. So the aim of this review is to provide a summary of self-report or patient-reported outcome measures which have been used to assess symptoms in pediatric narcoleptic patients, to investigate the current situation of the usage of these measures in pediatric narcolepsy assessment.

## Materials and methods

The searches of the literature for measures of symptoms of pediatric narcoleptic patients were performed in June 2018 in National Center for Biotechnology information (NCBI), the terms used were:”(questionnaire MeSH OR questionnaire OR diary OR diaries OR scale) AND narcolepsy MeSH” with no limits. “questionnaire MeSH OR questionnaire OR diary OR diaries OR scale) AND sleepiness”, “questionnaire MeSH OR questionnaire OR diary OR diaries OR scale) AND depression”, “questionnaire MeSH OR questionnaire OR diary OR diaries OR scale)AND ADHD”, “questionnaire MeSH OR questionnaire OR diary OR diaries OR scale) AND ( life quality OR health)”, “questionnaire MeSH OR questionnaire OR diary OR diaries OR scale) AND (sleep OR insomnia)”, with limits of 6–18 years old, English, abstract, and last 5 years.

Inclusion criteria were: 1) participants were human adolescents or children. 2) the questionnaires measuring symptoms or complications of narcolepsy were used in the study.

Repeated abstract were removed and abstracts were excluded if the population was not pediatric. Articles that mentioned a measure that seemed to be an effective questionnaire but did not give its name were reviewed in detail to determine the relevant questionnaires.

## Results

### The existing questionnaires for narcoleptic pediatric patients

#### Disease-driven questionnaires

The search for narcolepsy questionnaires returned 387 items, we found that only the narcolepsy severity scale [14] and the Ullanlinna Narcolepsy Scale [15] were narcolepsy-specific questionnaires. The narcolepsy severity scale was a brief, self-report questionnaire consisting of 15 items, it was developed and validated in a population of adult patients with narcolepsy type 1 to measure the severity of five major narcolepsy symptoms and assess the responsiveness to medications. The Ullanlinna Narcolepsy Scale is an 11-item scale assessing the two main features of the narcoleptic syndrome, the abnormal sleeping tendency and cataplexy. The Ullanlinna Narcolepsy Scale sum score reliably distinguishes patients with the narcoleptic syndrome from patients with sleep apnoea, multiple sclerosis and epilepsy [15]. However, both of them were developed in the adult population and none of them were validated in the pediatric population.

Recently, almost no disease-specific questionnaires exist in the field of pediatric patients with narcolepsy, which required further research of key interest.

#### Symptom-driven questionnaires

Although many questionnaires have been widely used to evaluate the primary symptoms and complications of narcoleptic children, neither of them was dedicated questionnaires for specific symptoms of narcolepsy nor has been validated in the pediatric population. If a scale measures two or more narcoleptic symptoms, it may be listed under two or more subheadings.

### Questionnaires for primary narcolepsy symptoms

#### Questionnaires for cataplexy

According to the literature searches, 35 abstracts were returned and reviewed. All cataplexy questionnaires were study-specific diaries and were not a validated questionnaire. A study reported the wording of their question, which is intended for adolescents [16]: “how frequently have you experienced episodes of sudden muscle weakness when you are having fun, excited, angry or laughing?” Most researches assessed cataplexy measured the frequency of attacks, which is accomplished through use of the study-specific diaries. A few studies measured severity, however, such assessments were based on arbitrary scales which have not been validated; for example, a study used an cataplexy severity rating scale (1 = minimal weakness, 2 = voluntarily preventable falls, 3 = falls to the ground) to measure the severity of cataplexy in pediatric patients with narcolepsy [17]. Another study used a four-point rating scale (1 = none, 2 = slightly improved, 3 = unchanged, 4 = clearly worsened) to evaluated cataplexy severity and frequency [18].

#### Questionnaires for sleepiness

The search for sleepiness questionnaires returned 492 items, after removing 64 duplicates, 14 reviews, 81 non-pediatric articles, the remained publications were kept as being used for the purpose of assessing EDS in pediatric population.

As presented in Tables 1, 20 questionnaires assess sleepiness in pediatric population. Of the 20 questionnaires assessing sleepiness in the pediatric population, the Epworth Sleepiness Score was most frequently used to measure sleepiness in pediatric population, followed by the Children’s Sleep Habits Questionnaire, Pediatric Daytime Sleepiness Scale and Pediatric Sleep Questionnaire. Epworth Sleepiness Score is a simple, self-administered questionnaire which is shown to provide a measurement of the subject’s general level of daytime sleepiness. It has been widely used to measure daytime sleep and sleepiness by researchers. The Epworth Sleepiness Score is based on questions referring to eight “most sleepy” situations. Subjects are asked to rate on a scale of 0–3 how likely they would be to doze off or fall asleep in the eight situations, based on their usual way of life in recent times. The numbers selected for the eight situations in the Epworth sleepiness score was added together to give a score for each subject, between 0 and 24 [12]. The Children’s Sleep Habits Questionnaire was developed in the USA. It is a parent-report, multidimensional questionnaire that was developed to evaluate the sleep behavior in school-aged children, detailed information of the Children’s Sleep Habits Questionnaire was in the nighttime sleep section. Pediatric Daytime Sleepiness Scale is widely used for assessing daytime sleepiness. It is a self-assessment instrument that describes some daily life situations related to sleep habits, waking time and sleep problems [19]. The entire questionnaire included 32 items related to daily sleep patterns, school achievement, mood, sleepiness, quality of life, and extracurricular activities. All questions were presented in a Likert-scale format. Based on the Likert-scale ratings, items were scored from 0 to 4. Higher scores indicates greater levels of sleepiness [20]. Among the scales evaluating sleepiness, Sleep Disturbance Scale for Children, [21] Pediatric Sleep Questionnaire, [22] and BEARS Questionnaire [23] were designed mainly for screening. Especially, The Pediatric Sleep Questionnaire was mostly used to assess children for sleep disordered breathing.

**Table 1:** Questionnaires for primary narcolepsy symptoms in the pediatric population

Questionnaire	Frequency of use
*Questionnaires for sleepiness*	
Epworth Sleepiness Scale and ESS-modified	50
Children’s Sleep Habits Questionnaire^a^	37
Pediatric Daytime Sleepiness Scale^a^	23
Pediatric Sleep Questionnaire^a^	20
Sleep Disturbance Scale for Children^a^	9
School Sleep Habits Survey^a^	6
Karolinska Sleepiness Scale	6
Sleep Disorder Scale for children^a^	3
ESS-CHAD*	2
Chidren’s Report of Sleep Patterns-Sleepiness Scale^a^	2
BEARS questionnaire	1
Children’s Sleep Hygiene Scale^a^	1
Clevaland Adolescent Sleepiness Questionnaire^a^	1
General Sleep Disturbance Scale	1
NIMHANS Sleep Disorders Questionnaire	1
Pictorial Sleepiness Scale	1
Sleepiness Scale adapted to Children and Adolescents^a^	1
Sleepiness Scale	1
Sleep-Wake Problems Behavior Scale	1
Sleep-Wake Habit Questionnaire	1
*Questionnaires for night sleep*	
Children’s Sleep Habits Questionnaire^a^	70
Pittsburgh Sleep Quality Index	38
Pediatric Sleep Questionnaire^a^	20
Sleep Disturbance Scale for Children^a^	9
Insomnia Severity Index	6
School Sleep Habits Survey^a^	6
Athens Insomnia Scale	4
Sleep Disorder Scale for Children^a^	3
Children’s Report of Sleep Patterns-Sleepiness Scale^a^	2
Adolescent Sleep Hygiene Scale^a^	2
Sleep Hygiene Questionnaire	2
Children’s Sleep Hygiene Scale^a^	1
Pre-sleep arousal scale	1
General Sleep Disturbance Scale	1
NIMHANS Sleep Disorders Questionnaire	1
Adolescent Sleep Wake Scale^a^	1
Sleep-Wake Problems Behavior Scale	1
Sleep-Wake Habit Questionnaire	1
Pediatric Sleep Disturbance Index^a^	1

*ESS* Epworth sleepiness score, *ESS-CHAD* Epworth sleepiness score for children and adolescents

^a^ Pediatric-specific questionnaires

#### Questionnaires for nighttime sleep

The search for Questionnaires for night sleep returned 2608 items, after removing 354 duplicates, 72 reviews, 424 non-pediatric articles, the remained publications were kept to identify the relevant questionnaires. If the information contained in the abstract is insufficient to determine the type of measures used, the complete articles were obtained. As shown in Table 1, the names of 19 questionnaires that included assessment of the quality of night sleep in the pediatric population were extracted from the relevant articles. The Children’s Sleep Habits Questionnaire was most frequently used to measure the quality of night sleep, followed by the Pittsburgh Sleep Quality Index, Pediatric Sleep Questionnaire, Sleep Disturbance Scale for Children, Insomnia Severity Index, School sleep Habits Survey and Athens Insomnia Scale For Children.

Of these questionnaires, the Children’s Sleep Habits Questionnaire was developed in the USA. It is a parent-report, multidimensional questionnaire that was developed to evaluate the sleep behavior in school-aged children [24]. It was designed as a screening tool with which to detect sleep problems for children, and is based on common clinical symptom presentations of the most prevalent pediatric International Classification of Sleep Disorders diagnoses [24, 25]. Pittsburgh Sleep Quality Index is a commonly used retrospective self-report questionnaire, that measures sleep quality over the previous month [26]. Seven clinically derived areas of sleep difficulties are assessed by the questionnaire, including sleep latency, sleep duration, habitual sleep efficiency, sleep quality, sleep disturbances, daytime dysfunction, and use of sleeping medications. It has been used frequently worldwide. The Pediatric Sleep Questionnaire consists of 4 parts: Breathing, Sleepiness, Behavior and others. This questionnaire has mainly been used in the patients with obstructive sleep apnea syndrome [22]. The Sleep Disturbance Scale for Children [21] is designed to assess six groups of common sleep disorders (sleep-disordered breathing, initiating and maintaining sleep, disorders of excessive somnolence, disorder of arousal, sleep-wake transition disorder, sleep hyperhidrosis) in pediatric population aged 6–15 years [27]. The Insomnia Severity Index is a brief instrument that assesses insomnia according to the criteria from the diagnostic and statistical manual of mental disorders-IV (DSM-IV) and the International Classification of Sleep Disorders [28]. The School sleep Habits Survey is a self-reported survey designed to assess the sleep/wake habits and typical daytime functioning of adolescents. The survey items queried students about usual sleeping and waking behaviors over the past surveys [29], which is a typical interval for waking behaviors over the past 2 weeks. As a thorough method for data collection, the survey allows researchers and clinicians alike to gather valuable demographic and behavioral information, including: sleep schedule regularity, school performance, daytime sleepiness, behavior problems, depressive mood, and bed times, rise times, and total sleep times for both weeknights and weekends [30]. The Athens Insomnia Scale estimates daytime and nocturnal sleep problems which occur at least three times per week according to the international classification of diseases-10 (ICD-10) criteria [31] and has been used to assess sleep problems of narcoleptic patients [32]. The Sleep Disorder Scale for Children, Children’s Report of sleep Patterns-Sleepiness Scale, Adolescent Sleep Hygiene Scale, Sleep Hygiene Questionnaire, Children’s Sleep Hygiene Scale, pre-sleep arousal Scale, General Sleep Disturbance Scale, NIMHANS Sleep Disorders Questionnaire, Adolescent Sleep Wake Scale were less frequently used according to the result of the literature searches, thus they were not considered for subsequent review.

### Questionnaires for comorbid narcolepsy symptoms

#### Questionnaires for depression

The first query for depression questionnaires returned 6025 items, after removing 743 duplicates, 72 reviews, 1216 non-pediatric articles, we found 11 measurement tools used as instruments for depression assessment among pediatric population, the names of them were shown in Table 2. The Children Depression Inventory, Center for Epidemiological Studies-Depression Scale and Depression Self-Rating Scale for Children were most frequently used to measure depression in the pediatric population, followed by The Depression Anxiety Stress Scale and Beck Depression Inventory. The Children’s Depression Inventory is a comprehensive assessment of depressive symptoms in pediatric population aged 7–17 years, [33] it divides depressive symptoms into five subscales: negative mood, ineffectiveness, anhedonia, interpersonal problems, and negative self-esteem. It has been used to evaluate the depressive symptoms of pediatric patients with narcolepsy [34]. The Center for Epidemiological Studies-Depression Scale and Beck depression inventory were usually compared to criterion standard diagnoses derived from diagnostic interview form [35, 36]. The Center for epidemiological studies depression scale is a questionnaire used world-wide to measure depressive symptoms, it is a 20-item tool developed to screen for depression in the general population, and has demonstrated good reliability and validity in a number of settings [35, 37]. The Depression Self-Rating Scale for Children is a typical example of self-rating scales designed to screen for pediatric depression. It makes fewer demands on cognitive abilities than the Children Depression Inventory, and it is a simple tool that can be used for young children [36, 38]. Base on these considerations, the Depression Self-Rating Scale for Children is frequently used in present researches [36, 38]. The Depression Anxiety Stress Scale is a self-report questionnaire. It was created with the initial purpose to differentiate the core symptoms between depression and anxiety [39]. The Beck depression inventory has been used in pediatric narcoleptic patients, the 21-item Beck depression inventory, first proposed by Beck et al., is one of the most popular measures of depressive symptoms worldwide, this instrument has been used in more than 7000 studies. The theoretical assumption of the original BDI relied upon the belief that negativistic distorted cognitions would be the core characteristic of depression [16, 40]. Other instruments measuring depression in the pediatric population were Zung Self-Rating Depression Scale, Beck Youth Inventory, Hamilton Depression Rating Scale, Adolescent Symptom Inventory-Depression Scale, Adolescent Depression Rating Scale and Kandel’s depressive symptoms scale for adolescents.

**Table 2:** Questionaires for comorbid narcolepsy symptoms for use in the pediatric population

Questionnaire	Frequency of use
*Questionnaires for depression*	
Children Depression Inventory^a^	107
Center for Epidemiological Studies-Depression Scale	55
Depression Self-Rating Scale for Children^a^	35
The Depression Anxiety Stress Scale	29
BDI-II	28
Zung Self-Rating Depression Scale	10
Beck Youth Inventory^a^	6
Hamilton Depression Rating Scale	6
Adolescent Symptom Inventory-Depression Scale^a^	2
Adolescent Depression Rating Scale^a^	1
Kandel’s depressive symptoms scale for adolescents^a^	1
*Questionnaires for attention-deficit/hyperactivity disorder*	
Child Behavior Checklist^a^	404
Strengths and Difficulties Questionnaire^a^	384
ADHD Rating Scale IV	73
Swanson, Nolan and Pelham Scale for parents^a^	39
ADHD self-report scale	13
Turgay DSM-IV-Based Child and Adolescent Behavioral Disorders^a^	13
Conners’ Parent Rating Scale-Revised^a^	8
The Wender Utah Rating Scale	4
Retrospective Modified Overt Aggression Scale	2
Knowledge of Attention Deficit Disorders Scale	2
Question prompt list	1
Child and Adolescent Disruptive Behavior Inventory^a^	1
ADHS-E	1

*BDI* Beck depression inventory, *ADHD* attention deficit and hyperactivity disorder, *ADHS-E* ADHD attention deficit and hyperactivity disorder-emotion&affect scale, *DSM-IV* diagnostic and statistical manual of mental disorders-IV

^a^Pediatric-specific questionnaires

#### Questionnaires for attention-deficit/hyperactivity disorder

The search for Questionnaires for attention-deficit/hyperactivity disorder (ADHD) returned 1245 items, after removing 134 duplicates, 26 reviews, 112 non-pediatric articles, the remained publications were kept as being used for the purpose of identifying relevant questionnaires assessing ADHD in pediatric population. We extracted the names of questionnaires that included assessment of the ADHD symptoms in the pediatric population. Of the 13 questionnaires assessing ADHD in the pediatric population, as shown in Table 2, the Child Behavior Checklist has been widely used for assessing emotional and behavioral problems in the pediatric population [41]. It consists of 2 broad scales (externalizing and internalizing scale) and 8 subscales. The subscales withdrawn, somatic complaints, and anxiety belong to the internalizing scale, the rule-breaking behavior and aggressive behavior are included in the externalizing scale [41]. Other questionnaires that frequently used were Strengths and Difficulties Questionnaire, ADHD Rating Scale IV, Swanson, Nolan and Pelham Scale for parents, ADHD self-report scale, Turgay DSM-IV-Based Child and Adolescent Behavioral Disorders. The Strengths and Difficulties Questionnaire is a measure of mental health problems of children aged 4–17, which can be used by parents/carers, teachers and children aged 11 or over. It contains 20 items relating to conduct problems, emotional symptoms, peer problems and hyperactivity [42]. The ADHD Rating Scale IV contains 18 items derived directly from the DSM-IV diagnostic criteria. Exploratory factor analysis showed that the two factors of ADHD impulse were similar to the the DSM-IV classification of ADHD. It is a behavior rating scale with good psychometric characteristics and is widely used to measure ADHD symptoms in school-age children [43]. The Swanson, Nolan and Pelham Scale consists of the DSM-IV symptoms of inattention and the hyperactivity/impulsivity ADHD criteria, and the opposite symptoms of defiant disorder criteria. The three subscales of the the Swanson, Nolan and Pelham scale proved to be the most sensitive way to record significant differences between treatment groups in the primary analyses (the multimodal treatment study of children with ADHD (MTA) Cooperative Group, 1999), which has been used in some clinical trials to assess the efficacy of treatment for ADHD [44, 45]. The ADHD Self-Report Scale includes the 18 symptom items, and ADHD is defined according to the DSM-IV-TR and the 5th edition of the DSM. It is divided into two parts, Part A represents symptoms of inattention, and part B represents symptoms of hyperactivity and impulsivity. The total score of ADHD self-report scale has good reliability and validity in both clinical and population samples [46, 47]. The Turgay DSM-IV-Based Child and Adolescent Behavioral Disorders was developed by Turgay according to DSM-IV diagnostic criteria, including 9 items regarding attention deficit, 3 items regarding impulsivity, 8 items regarding oppositional defiant disorder, 6 items regarding hyperactivity, and 15 items regarding conduct disorder [48]. Other less frequently used questionnaires that can be used to measure ADHD symptoms in pediatric population includes the Conners’ Parent Rating Scale-Revised, the Wender Utah Rating Scale, Retrospective Modified Overt Aggression Scale, Knowledge of Attention Deficit Disorders Scale, Question Prompt List, Child and Adolescent Disruptive Behavior Inventory and ADHD attention deficit and hyperactivity disorder-emotion&affect scale (ADHS-E).

#### Questionnaires for life quality

The query for Life quality questionnaires returned 205 items, after removing 6 reviews, 42 non-pediatric articles, we found 15 measurement tools used as instruments for life quality assessment among pediatric population (Table 3). Of the 15 questionnaires, KIDSCREEN, Children Health Questionnaire, Pediatric Quality of Life Inventory, the Quality of Life Health survey short-form-36 health survey (SF-36) and the Childhood Health Assessment Questionnaire were most frequently used. The KIDSCREEN is a validated measure of the construct for pediatric population. The KIDSCREEN-52 measures 10 facets on health related quality of life (HRQoL), including physical well-being, psychological well-being, self perception, moods and emotions, autonomy, parent relation and home life, peers and social support, financial resources, school environment, bullying [49], while a shortened version KIDSCREEN-27 measures 5 facets of HRQoL (physical well-being, psychological well-being, autonomy and parent relation, peers and social support, school environment) [50]. The Children Health Questionnaire was developed for children and according to the World Health Organization definition of health as a state of complete mental, physical, and social well-being and not merely the absence of infirmity or disease [51]. The 23-item Pediatric Quality of Life Inventory was used to evaluate HRQoL, it assesses the problem frequency within the domains of physical, social, emotional and school functioning [52]. It has been used to evaluate the life quality of narcoleptic patients [53]. The SF-36 was developed to measure “general health concepts not specific to any age, disease, or treatment group” [54]. The questionnaire assesses the following health concepts: limitations in social activities because of physical or emotional problems, limitations in physical activities because of health problems, limitations in usual role activities because of emotional problems, general health perceptions and vitality [54]. The questionnaire has been used to evaluate the impact of narcolepsy on the life quality of patients [55]. The Childhood health assessment questionnaire is one of the most widely used self-report questionnaires to measure function status in pediatric patients, it consisted of 36 items, measuring eight functional areas: dressing and grooming arising eating, walking, hygiene, reach, grip and activities [56, 57]. Other instruments are less frequently used in the assessment of life quality in the pediatric population, they are HRQoL Questionnaire, General Well-Being Scale, General Health Rating Index, Health Utility Index-2, Youth Generic Quality of Life Scale, EuroQol five dimensions-youth, International quality of life-Adolescents, Generic Children’s quality of life (QoL) Measure, Lansky play performance Scale and the Narcolepsy quality-of-life instrument. The Narcolepsy quality-of-life instrument with 21 questions [58] was applied to assess the quality of life in patients with narcolepsy [59].

**Table 3:** Life quality questionnaires for use in the pediatric population

Questionnaire	Frequency of use
KIDSCREEN^a^	128
Children Health Questionnaire^a^	71
Pediatric Quality of Life Inventory^a^	63
the Quality of Life Health Survey SF-36	20
Childhood Health Assessment Questionnaire^a^	16
HRQoL questionnaire	10
General Well-Being Scale	8
General Health Rating Index	7
Health Utility Index-2	4
Youth Generic Quality of Life Scale^a^	3
NARQoL-21^a^	2
EQ-5D-Y^a^	1
IQQOL-Adolescents^a^	1
Generic Children’s QoL Measure^a^	1
Lansky play performance scale^a^	1

*SF-36* short-form-36 health survey, *HRQoL* health related quality of life, *EQ-5D-Y* EuroQol five dimensions (youth), *IQQOL* International quality of life; *QoL* quality of life, *NARQoL-21* Narcolepsy quality of life-21

^a^Pediatric-specific questionnaires

### What questionnaires are still missing for narcoleptic pediatric patients

There are pediatric-specific questionnaires measuring the severity of sleepiness and disrupted nighttime sleep in pediatric narcolepsy patients, such as Pediatric Daytime Sleepiness Scale and Sleep Disturbance Scale for Children. As for the measurement of comorbid narcolepsy symptoms, there are pediatric-specific questionnaires to measure depression and attention-deficit/hyperactivity disorder, as well as the life quality of narcoleptic children. As for the measurement of cataplexy, however, all the cataplexy questionnaires were not identifiable as recognized validated questionnaires. Moreover, although the questionnaires mentioned above can measure the severity of narcolepsy symptoms, they are non-specific for symptoms in the scope of narcolepsy. Therefore, the absence of dedicated questionnaires for specific symptoms of narcolepsy is still an important problem to be solved. There is also a lack of validated narcolepsy-specific questionnaires for diagnosis and symptom severity assessment. Recently, the narcolepsy-specific questionnaires, narcolepsy severity scale and Ullanlinna Narcolepsy Scale were developed and have only been validated in a population of adult narcoleptic patients, so there is a lack of narcolepsy-specific questionnaires for pediatric narcolepsy patients. Moreover, a real patient- reported outcome narcolepsy-specific questionnaire is urgently needed.

## Discussion

Although a number of screening, diagnostic, and treatment-monitoring measures have been developed and evaluated for narcolepsy, it is notable now little attention has been given to addressing their efficacy and utility for use in the pediatric population. There are many questionnaires have been used to by researchers to assess symptoms of pediatric narcoleptic patients, but none of them has been validated in pediatric narcoleptic patients. Many researchers even use adult questionnaires to evaluate the symptom severity of pediatric narcoleptic patients, and ask parents/caregivers or other proxy responders to answer for children unable to understand the instruments. This factor introduces a number of variables and uncertainty that could affect accuracy of reporting.

Thus, measures are needed that not only can be administered to pediatric patients but have also been validated for use by proxies. Although measures used in clinical practice might vary from those used in academic research, this review confirms the shortage of validated questionnaires available for assessing common symptoms and complications (EDS, cataplexy, depression, night sleep) in pediatric narcoleptic patients, especially the lack of narcolepsy-specific questionnaires for use in the pediatric population. According to recent researches, there is also an unmet need for standardized and easily administered instruments for screening/diagnosis of pediatric narcolepsy, as well as to evaluate the effect of treatment over time with application for both clinical practice and clinical studies.

The need can be filled by adaptation or modification of existing measures that are applied in adults, such as the Ullanlinna Narcolepsy Scale and narcolepsy severity scale, to make them child-friendly. Using child-friendly language may present the most appropriate approach for assessing pediatric narcoleptic symptoms. Moreover, to overcome this limitation, the pediatric version of Ullanlinna Narcolepsy Scale and narcolepsy severity scale, as well as other new questionnaires should be developed for children to help diagnosis and assessing symptom severity in pediatric narcoleptic patients. Finally, the existing narcolepsy-specific questionnaires were not patient-reported outcome questionnaires, which may affect the accuracy of the measurement of narcoleptic symptoms. Therefore, a real patient-reported outcome questionnaire oriented toward pediatric narcoleptic patients should be provided in the future.

## Conclusion

In conclusion, our results suggest the need for developing standardized and easily administered instruments for diagnosis and assessing symptom severity in pediatric patients with narcolepsy. This need can be filled by adaptation or modification of existing measures that are applied in adults and developing patient-reported outcome questionnaires oriented toward pediatric population in the future.

## Data Availability

Not applicable.

## Abbreviations

ADHD: Attention deficit and hyperactivity disorder
ADHS-E: ADHD attention deficit and hyperactivity disorder-emotion&affect scale
BDI: Beck depression inventory
DSM-IV: Diagnostic and statistical manual of mental disorders-IV
EDS: Excessive daytime sleepiness
ESS: Epworth sleepiness score
ESS-CHAD: Epworth sleepiness score for children and adolescents
EQ-5D-Y: EuroQol five dimensions-youth
HRQoL: health related quality of life
IQQOL-Adolescents: International quality of life-adolescents
ICD-10: International classification of diseases-10
MTA: Multimodal treatment study of children with ADHD
NCBI: National center for biotechnology information
NARQoL-21: Narcolepsy quality of life-21
QoL: Quality of life
SF-36: Short-form-36 health survey

## References

[1] Partinen M, Kornum BR, Plazzi G, Jennum P, Julkunen I, Vaarala O (2014). Narcolepsy as an autoimmune disease: the role of H1N1 infection and vaccination. Lancet Neurol.

[2] Dauvilliers Y, Arnulf I, Mignot E (2007). Narcolepsy with cataplexy. Lancet (London, England).

[3] Thorpy MJ (2006). Cataplexy associated with narcolepsy: epidemiology, pathophysiology and management. CNS Drugs.

[4] Lecendreux M, Lavault S, Lopez R, Inocente CO, Konofal E, Cortese S, Franco P, Arnulf I, Dauvilliers Y (2015). Attention-deficit/hyperactivity disorder (ADHD) symptoms in pediatric narcolepsy: a cross-sectional study. Sleep..

[5] Roth T, Dauvilliers Y, Mignot E, Montplaisir J, Paul J, Swick T, Zee P (2013). Disrupted nighttime sleep in narcolepsy. J Clin Sleep Med.

[6] Ohayon MM, Ferini-Strambi L, Plazzi G, Smirne S, Castronovo V (2005). Frequency of narcolepsy symptoms and other sleep disorders in narcoleptic patients and their first-degree relatives. J Sleep Res.

[7] Ohayon MM (2013). Narcolepsy is complicated by high medical and psychiatric comorbidities: a comparison with the general population. Sleep Med.

[8] Zamarian L, Hogl B, Delazer M, Hingerl K, Gabelia D, Mitterling T (2015). Subjective deficits of attention, cognition and depression in patients with narcolepsy. Sleep Med.

[9] Stores G, Montgomery P, Wiggs L (2006). The psychosocial problems of children with narcolepsy and those with excessive daytime sleepiness of uncertain origin. Pediatrics..

[10] Jennum P, Ibsen R, Petersen ER, Knudsen S, Kjellberg J (2012). Health, social, and economic consequences of narcolepsy: a controlled national study evaluating the societal effect on patients and their partners. Sleep Med.

[11] Dodel R, Peter H, Spottke A, Noelker C, Althaus A, Siebert U, Walbert T, Kesper K, Becker HF, Mayer G (2007). Health-related quality of life in patients with narcolepsy. Sleep Med.

[12] Johns MW (1991). A new method for measuring daytime sleepiness: the Epworth sleepiness scale. Sleep..

[13] Mitler MM, Gujavarty KS, Browman CP (1982). Maintenance of wakefulness test: a polysomnographic technique for evaluation treatment efficacy in patients with excessive somnolence. Electroencephalogr Clin Neurophysiol.

[14] Dauvilliers Y, Beziat S, Pesenti C, Lopez R, Barateau L, Carlander B, Luca G, Tafti M, Morin CM, Billiard M, Jaussent I (2017). Measurement of narcolepsy symptoms: the narcolepsy severity scale. Neurology..

[15] Hublin C, Kaprio J, Partinen M, Koskenvuo M, Heikkila K (1994). The Ullanlinna narcolepsy scale: validation of a measure of symptoms in the narcoleptic syndrome. J Sleep Res.

[16] Lee YJ, Cho SJ, Cho IH, Jang JH, Kim SJ (2012). The relationship between psychotic-like experiences and sleep disturbances in adolescents. Sleep Med.

[17] Murali H, Kotagal S (2006). Off-label treatment of severe childhood narcolepsy-cataplexy with sodium oxybate. Sleep..

[18] Huang YS, Guilleminault C, Chen CH, Lai PC, Hwang FM (2014). Narcolepsy-cataplexy and schizophrenia in adolescents. Sleep Med.

[19] Meyer C, Ferrari GJJ, Barbosa DG, Andrade RD, Pelegrini A, PG FÉ (2017). Analysis of daytime sleepiniess in adolescents by the Pediatric daytime sleepiness scale: a systematic review. Rev Paul Pediatr.

[20] Drake C, Nickel C, Burduvali E, Roth T, Jefferson C, Pietro B (2003). The pediatric daytime sleepiness scale (PDSS): sleep habits and school outcomes in middle-school children. Sleep..

[21] Bruni O, Ottaviano S, Guidetti V, Romoli M, Innocenzi M, Cortesi F (1996). The sleep disturbance scale for children (SDSC). Construction and validation of an instrument to evaluate sleep disturbances in childhood and adolescence. J Sleep Res.

[22] Chervin RD, Hedger K, Dillon JE, Pituch KJ (2000). Pediatric sleep questionnaire (PSQ): validity and reliability of scales for sleep-disordered breathing, snoring, sleepiness, and behavioral problems. Sleep Med.

[23] Owens JA, Dalzell V (2005). Use of the 'BEARS' sleep screening tool in a pediatric residents' continuity clinic: a pilot study. Sleep Med.

[24] Owens JA, Spirito A, McGuinn M, Nobile C (2000). Sleep habits and sleep disturbance in elementary school-aged children. J Dev Behav Pediatr.

[25] Sun WQ, Chen WJ, Jiang YR, Li F, Li SH, Yan CH (2012). The association of sleep hygiene and sleep quality among school-age children. Zhonghua Yu Fang Yi Xue Za Zhi.

[26] Buysse DJ, Reynolds CF, Monk TH, Berman SR, Kupfer DJ (1989). The Pittsburgh sleep quality index: a new instrument for psychiatric practice and research. Psychiatry Res.

[27] Saffari M, Gholamrezaei A, Saneian H, Attari A, Bruni O (2014). Linguistic validation of the sleep disturbance scale for children (SDSC) in Iranian children with Persian language. Sleep Med.

[28] Gagnon C, Belanger L, Ivers H, Morin CM (2013). Validation of the insomnia severity index in primary care. J Am Board Fam Med.

[29] Malloggi S. Conte F, Prevalence and determinants of bad sleep perception among italian children and adolescents. Int J Environ Res Public Health. 2020;17(24):9363. 10.3390/ijerph17249363. 10.3390/ijerph17249363 PMC7765082 33327567

[30] Olorunmoteni OE, Fatusi AO, Komolafe MA, Omisore A (2018). Sleep pattern, socioenvironmental factors, and use of electronic devices among Nigerian school-attending adolescents. Sleep health.

[31] Okajima I, Nakajima S, Kobayashi M, Inoue Y (2013). Development and validation of the Japanese version of the Athens insomnia scale. Psychiatry Clin Neurosci.

[32] Walacik-Ufnal E, Piotrowska AJ, Wolynczyk-Gmaj D, Januszko P, Gmaj B, Ufnal M (2017). Narcolepsy type 1 and hypersomnia associated with a psychiatric disorder show different slow wave activity dynamics. Acta Neurobiol Exp.

[33] Kovacs M (1985). The Children's depression, inventory (CDI). Psychopharmacol Bull.

[34] Inocente CO, Gustin MP, Lavault S, Guignard-Perret A, Raoux A, Christol N, Gerard D, Dauvilliers Y, Reimão R, Bat-Pitault F, Lin JS, Arnulf I, Lecendreux M, Franco P (2014). Depressive feelings in children with narcolepsy. Sleep Med.

[35] Chhabria KS, Carnaby GD (2017). Psychometric validation of the Center for Epidemiological Studies Depression Scale in head and neck Cancer patients. Oral Oncol.

[36] Birleson P, Hudson I, Buchanan DG, Wolff S (1987). Clinical evaluation of a self-rating scale for depressive disorder in childhood (depression self-rating scale). J Child Psychol Psychiatry Allied Discip.

[37] Betancourt T, Scorza P, Meyers-Ohki S, Mushashi C, Kayiteshonga Y, Binagwaho A, Stulac S, Beardslee WR (2012). Validating the Center for Epidemiological Studies Depression Scale for children in Rwanda. J Am Acad Child Adolesc Psychiatry.

[38] Denda K, Kako Y, Kitagawa N, Koyama T (2006). Assessment of depressive symptoms in Japanese school children and adolescents using the Birleson depression self-rating scale. Int J Psychiatry Med.

[39] Lovibond PF, Lovibond SH (1995). The structure of negative emotional states: comparison of the depression anxiety stress scales (DASS) with the Beck depression and anxiety inventories. Behav Res Ther.

[40] Wang YP, Gorenstein C (2013). Psychometric properties of the Beck Depression Inventory-II: a comprehensive review. Rev Bras Psiquiatr.

[41] Shelton AR, Malow B (2017). Correlates to problem behaviors in pediatric narcolepsy: a pilot study. J Clin Sleep Med.

[42] Goodman R (2001). Psychometric properties of the strengths and difficulties questionnaire. J Am Acad Child Adolesc Psychiatry.

[43] López-Villalobos JA, Andrés-De Llano J, López-Sánchez MV, Rodríguez-Molinero L, Garrido-Redondo M, Sacristán-Martín AM (2017). Criterion validity and clinical usefulness of attention deficit hyperactivity disorder rating scale IV in attention deficit hyperactivity disorder (ADHD) as a function of method and age. Psicothema..

[44] Abikoff H, McGough J, Vitiello B, McCracken J, Davies M, Walkup J, Riddle M, Oatis M, Greenhill L, Skrobala A, March J, Gammon P, Robinson J, Lazell R, McMahon D, Ritz L, RUPP ADHD/Anxiety Study Group (2005). Sequential pharmacotherapy for children with comorbid attention-deficit/hyperactivity and anxiety disorders. J Am Acad Child Adolesc Psychiatry.

[45] Correia Filho AG, Bodanese R, Silva TL, Alvares JP, Aman M, Rohde LA (2005). Comparison of risperidone and methylphenidate for reducing ADHD symptoms in children and adolescents with moderate mental retardation. J Am Acad Child Adolesc Psychiatry.

[46] Adler LA, Spencer T, Faraone SV, Kessler RC, Howes MJ, Biederman J, Secnik K (2006). Validity of pilot adult ADHD self- report scale (ASRS) to rate adult ADHD symptoms. Ann Clin Psychiatry.

[47] van de Glind G, van den Brink W, Koeter MW, Carpentier PJ, van Emmerik-van Oortmerssen K, Kaye S, Skutle A, Bu ET, Franck J, Konstenius M, Moggi F, Dom G, Verspreet S, Demetrovics Z, Kapitány-Fövény M, Fatséas M, Auriacombe M, Schillinger A, Seitz A, Johnson B, Faraone SV, Ramos-Quiroga JA, Casas M, Allsop S, Carruthers S, Barta C, Schoevers RA, Levin FR, IASP Research Group (2013). Validity of the adult ADHD self-report scale (ASRS) as a screener for adult ADHD in treatment seeking substance use disorder patients. Drug Alcohol Depend.

[48] Ayaz M, Ayaz AB, Soylu N, Yuksel S (2014). Medication persistence in Turkish children and adolescents with attention-deficit/hyperactivity disorder. J Child Adolesc Psychopharmacol.

[49] Ravens-Sieberer U, Gosch A, Rajmil L, Erhart M, Bruil J, Duer W, Auquier P, Power M, Abel T, Czemy L, Mazur J, Czimbalmos A, Tountas Y, Hagquist C, Kilroe J, KIDSCREEN Group E (2005). KIDSCREEN-52 quality-of-life measure for children and adolescents. Expert Rev Pharmacoeconomics Outcomes Res.

[50] Ravens-Sieberer U, Auquier P, Erhart M, Gosch A, Rajmil L, Bruil J (2007). The KIDSCREEN-27 quality of life measure for children and adolescents: psychometric results from a cross-cultural survey in 13 European countries. Qual Life Res.

[51] Fayed N, Cieza A, Bickenbach J (2012). Illustrating child-specific linking issues using the child health questionnaire. Am J Phys Med Rehabil.

[52] Varni JW, Seid M, Kurtin PS (2001). PedsQL 4.0: reliability and validity of the pediatric quality of life inventory version 4.0 generic core scales in healthy and patient populations. Med Care.

[53] Rocca FL, Finotti E, Pizza F, Ingravallo F, Gatta M, Bruni O, Plazzi G (2016). Psychosocial profile and quality of life in children with type 1 narcolepsy: a case-control study. Sleep..

[54] Ware JE, Sherbourne CD (1992). The MOS 36-item short-form health survey (SF-36). I. Conceptual framework and item selection. Med Care.

[55] Kapella MC, Berger BE, Vern BA, Vispute S, Prasad B, Carley DW (2015). Health-related stigma as a determinant of functioning in young adults with narcolepsy. PLoS One.

[56] Miyamae T, Tani Y, Kishi T, Yamanaka H, Singh G (2020). Updated version of Japanese childhood health assessment questionnaire (CHAQ). Mod Rheumatol.

[57] Pouchot J, Ecosse E, Coste J, Guillemin F (2004). Validity of the childhood health assessment questionnaire is independent of age in juvenile idiopathic arthritis. Arthritis Rheum.

[58] Chaplin JE, Szakacs A, Hallbook T, Darin N (2017). The development of a health-related quality-of-life instrument for young people with narcolepsy: NARQoL-21. Health Qual Life Outcomes.

[59] Becker PM, Schwartz JR, Feldman NT, Hughes RJ (2004). Effect of modafinil on fatigue, mood, and health-related quality of life in patients with narcolepsy. Psychopharmacology.

